# Software is Policy: Electronic Health Record Governance and the Implications of Clinical Standardization

**DOI:** 10.1007/s11606-023-08280-7

**Published:** 2023-10-05

**Authors:** Julian Brunner, Shay Cannedy, Matthew McCoy, Alison B. Hamilton, Jeremy Shelton

**Affiliations:** 1 Center for the Study of Healthcare Innovation, Implementation & Policy, VA Greater Los Angeles Healthcare System, Los Angeles, CA USA; 2 Department of Psychiatry and Biobehavioral Sciences, David Geffen School of Medicine, University of California Los Angeles, Los Angeles, CA USA; 3 George E. Wahlen Veterans Affairs Medical Center, Salt Lake City, UT USA; 4 Department of Urology, David Geffen School of Medicine, University of California Los Angeles, Los Angeles, CA USA

**Keywords:** electronic health record transition, standardization, centralization, implementation, qualitative research, veterans

## Abstract

**Background:**

Electronic health record (EHR) implementations, whether replacing paper or electronic systems, are major social and organizational transformations. Yet studies of EHR-to-EHR transitions have largely neglected to elucidate accompanying social and organizational changes. One such underexplored change is the standardization of clinical practice in the context of EHR transitions.

The Department of Veterans Affairs (VA) has begun a decade-long process of replacing the approximately 130 separate versions of its homegrown EHR with a single commercial EHR system. This provides an opportunity to explore the standardization of clinical practice amidst an EHR transition.

**Objective:**

To identify, in the context of a large-scale EHR transition, (1) the scope and content of clinical standardization and (2) the anticipated implications of such standardization.

**Design:**

Qualitative study.

**Participants:**

Twenty-nine members of VA councils established for the EHR transition.

**Approach:**

We conducted semi-structured interviews, which were professionally transcribed, and analyzed first using rapid analysis methods, followed by coding and content analysis.

**Key Results:**

Clinical standardization across facilities was a central goal of the EHR transition, encompassing computerized recommendations, order sets, professional roles/permissions, and clinical documentation. The anticipated *implications* of this standardization include (i) potential efficiency gains, with less duplicated effort across facilities; (ii) expanded bureaucracy; and (iii) increased uniformity, reducing both wanted and unwanted variation in care.

**Conclusions:**

EHR systems shape a wide range of clinical processes, particularly in a large organization like VA with a long history of EHR use. This makes standardization of EHR content a powerful mechanism for standardizing clinical practice itself, which can bring dramatic collateral consequences. Organizations undergoing EHR transitions need to recognize the important role that clinical standardization plays by treating EHR transitions as major organizational transformations in the governance of clinical practice.

**Supplementary Information:**

The online version contains supplementary material available at 10.1007/s11606-023-08280-7.

## INTRODUCTION

The implementation of a new electronic health record (EHR) system, whether replacing paper records or a prior EHR system, is a deeply *social and organizational transformation*. This long-appreciated feature of EHR implementation is the reason that several prominent theoretical frameworks in health IT take a *sociotechnical* approach that considers interactions among the technology and the organization’s “existing social and technical systems.”^1–3^ One early proponent of a sociotechnical approach to understanding EHR implementation suggested that “getting such technologies to work in concrete health care practices appears to be a politically textured process of organizational change”^4^ and later suggested that “overlooking the fact that [EHR] implementation will fundamentally affect the health care organization’s structures and processes is one core reason for implementation failure.”^5^ Yet literature on increasingly common EHR-to-EHR transitions barely reflects this reality. Studies of EHR transitions have shed light on the challenges of data migration, the technical capabilities typically lost or gained, and the average valence, magnitude, and change over time in EHR-related outcomes such as user satisfaction and patient safety.^6–9^ In other words, they have been studied as information technology (IT) projects, and assessed on the basis of whether they were positive or negative in a variety of domains, with relatively little attention paid to surfacing the social and organizational changes that are typical of even smaller-scale implementations of health IT.^10^

Social and organizational changes related to smaller-scale health IT implementations are countless, and many have been well-documented. For example, computerized provider order entry (CPOE) has been linked with striking changes in clinical team members’ relative power, control, and autonomy.^11^ Doctor-patient communication^12^ and care coordination^13^ are often dramatically altered by health IT implementation.

One such organizational change related to health IT implementation is the standardization of clinical practice. Implementation of health IT often seeks to standardize previously heterogeneous clinical practices by “hardwiring the standards into the EHR system,” as described in a study of an EHR implementation in Denmark.^14^ However, beyond that study’s acknowledgement of “two related tensions: between standardization and adaptation, and between centralized control and local autonomy,” the role of clinical standardization in EHR transitions has been remarkably underexplored.

To address this gap, this study explores the role of standardization in an ongoing large-scale EHR transition at the United States Department of Veterans Affairs (VA). The VA has begun a roughly decade-long process of replacing the approximately 130 separate versions of its homegrown EHR with a single system developed by the Oracle Cerner Corporation whose core structure and features will be shared not only across all VA facilities, but also across all Defense Health Agency (DHA, formerly Department of Defense) facilities. In doing so, it will be the first healthcare organization of its scale to make this transformation from a federated (also called “distributed”) model, in which EHR content decisions are primarily made at individual hospitals or small regional networks, to a centralized model, in which most EHR content decisions are made nationally on behalf of hundreds of facilities.

In this study, we draw upon interviews with VA employees most directly involved with the development and configuration of the VA’s new EHR, using findings from these interviews to address two key questions: (1) what is the scope and content of clinical standardization (i.e., How wide-reaching is it? What kinds of clinical processes are subject to standardization?); and (2) what are the anticipated implications of such standardization? By providing a better understanding of the scope, content, and implications of standardization in the context of an EHR transition, we hope to inform future EHR transition efforts, as well as other initiatives involving the standardization of clinical practices.

## METHODS

### Study Population

As a central part of the process of configuring the Oracle Cerner EHR for VA, national “EHR modernization councils” (hereafter, “councils”) of VA employees were established in 2019 to guide the content and overall design of the EHR. Councils were organized around different aspects of care delivery and clinical specialties, and were intended to approximately correspond to individual components, or “solutions” within the Oracle Cerner EHR platform. Councils included a mix of subject matter experts, field-based clinician representatives, and representatives from national VA offices, working with a team of consultants and Oracle Cerner representatives. We interviewed members of these councils with the goal of better understanding the role of the councils in the EHR transition, the changing nature of EHR governance, and key opportunities and challenges in the transition and the governance of the EHR.

Semi-structured telephone interviews were conducted with council members, emphasizing perspectives from council chairs, leaders of workgroups within councils, and other council members who were particularly involved, as identified by other participants or by the VA office that established and managed the councils. All interview participants were VA employees; most were physicians, nurses, and allied health professionals with clinical responsibilities alongside official regional or national leadership roles relevant to their respective council. In order to focus on EHR governance most proximate to clinical care, we limited our study to the 12 clinically focused councils (e.g., ambulatory care, perioperative care, acute care delivery, behavioral health) and did not interview members of the 6 councils responsible for other aspects of the enterprise (e.g., business operations, supply chain). Due to heterogeneity in council size and scope (e.g., the ambulatory council included 32 members across 7 workgroups, while the dentistry council included 8 members across 2 workgroups), we recruited more participants from larger councils when possible (Table 1).

**Table 1:** EHR Councils and Interview Participants Recruited

Council name	Council size*	Interview participants
Ambulatory	32	6
Perioperative Care	25	4
Acute Care Delivery	37	3
Behavioral Health	17	3
Acute Provider	14	2
Emergency Medicine	18	2
Clinical Support Services	9	2
Dentistry	8	2
Pharmacy	23	2
Geriatrics and Extended Care	11	1
Patient Engagement and Virtual Health	7	1
Rehabilitation and Acute Clinical Ancillaries	15	1

^*^At the time of recruitment

### Data Collection and Management

Twenty-nine interviews were conducted from July to December 2020. Each approximately 60-min interview was conducted by an experienced qualitative interviewer (SC, MM, or JB), with at least one other interviewer present to take notes and ensure that interviews addressed all intended topics. The interview guide (online appendix) was organized around the goal of understanding the nature of the councils’ work and their role in the EHR transition. We built upon Donabedian’s “structure, process, outcome” model,^15^ and integrated principles of formative evaluation to develop an interview guide that would give us insight into the structure, processes, and outcomes of the councils, as well as additional information about their inputs, lessons learned, and future directions. The guide covered six key topics: (1) council structure, scope, and composition; (2) council processes (e.g., content of council meetings and activities, communication within and across councils, team dynamics); (3) inputs to council decision-making (information sources drawn upon); (4) council outputs (EHR and clinical content produced by the council); (5) lessons and advice; and (6) perspectives about EHR governance at later stages of implementation/sustainment. Interviews were recorded and transcribed.

### Analysis

We used a sequence of rapid qualitative analysis (e.g., matrix analysis) followed by content analysis methods to guide our review of the data.^16–18^ First, we created a structured summary template that was used by our team to summarize interview data from each interview, identifying key points raised in interviews related to each of the primary domains of interest in our study (the topics covered in our interview guide). Next, the analytic team created a “summary of summaries” matrix that consolidated findings across interviews and captured key points and themes. A three-person coding team (SC, MM, JB) reviewed these structured summaries to generate an initial list of codes. We used ATLAS.ti to organize our coding, guided by content analysis principles.^18^ The code list was iteratively refined and discrepancies in coding were resolved through consensus. This analysis identified clinical standardization and its implications as key emergent phenomena. To understand these phenomena further, the lead author identified and compiled themes and sub-themes related to EHR standardization and worked with a co-author (SC) to identify associated quotes from transcripts. To ensure thoroughness of our interpretations, we examined negative cases as we were developing the themes, and while we cannot assert that nothing new was being learned in the final interviews, we were at that point hearing consistent information that contributed to our confidence about the results presented in the paper. This study was approved by the local VA Greater Los Angeles Institutional Review Board.

## RESULTS

Below, we first describe the scope and content of standardization, offering examples of the EHR configuration decisions that councils were asked to make on behalf of all VA facilities. Then, we describe key *implications* of clinical standardization identified by participants — i.e., important and potentially underappreciated likely consequences of clinical standardization via EHR. Selected segments of quotations that best illustrate key points are shown in bold. Because the Cerner Corporation was acquired by Oracle after the interviews were conducted, the company and product now known as “Oracle Cerner” is referred to by participants as “Cerner.”

### Software is Policy: Scope and Content of Standardization

#### Scope of Standardization

While our interview guide did not include questions about the underlying motivation for the EHR transition or the role of standardization, several participants volunteered that standardization was a central and intentional goal of the EHR implementation. One participant noted that “**one of the big goals of this whole project is** to achieve a much higher degree of **standardization across the enterprise**.” (ID24) Another articulated the impact on clinical care more directly, explaining, “so **what they want is to have one single approach** that every VA in the country uses for its clinical care delivery,” and elaborating, “**part of the goal** of this whole transition that was set by national in making this transition **was to enforce standardization across all VAs**. The perception was that the lack of standardization … is absolutely, inherently a problem.” (ID13).

#### Content of Standardization

In addition to describing the large role that standardization was playing in the implementation, participants conveyed the breadth of clinical practice that would be shaped by the newly standardized EHR. In several cases, this included identifying which interventions would be recommended or facilitated by the EHR. One participant gave an example in primary care:

We had to **review all of the health maintenance recommendations** and work on sort of developing the … **order associated with that recommendation** so you can complete it … Cerner has some in their [default version] and we have to make sure that those sort of **agree with VA policy**. (ID10)

In other cases, rather than identifying interventions that would be explicitly recommended by the EHR, the councils were charged with choosing which approaches to care to encourage by making them easily accessible and convenient.

We’ve been **curating orders** that were going to go on their quick views, quick visits. A lot of it was about having things set up so that if [for example] you were seeing someone for like a urinary tract infection, you’ve got the **antibiotics at the right dosage**. (ID03)

In configuring the EHR, council members also described how their decisions would delineate the roles and permissions of each member of a clinical team. The same participant who described curating orders also explained the implications of council work on “identity management,” which they described as, “What are the different roles that are there and **what are the authority mixes of, you know, “Who can prescribe meds?**” “Who can do this?”(ID03) Another participant underscored the implications of identity management by explaining how much depends on the individual permissions granted to each EHR user, in contrast to the less regimented approach of the VA’s homegrown EHR:

The way [Oracle Cerner’s] workflows work is they **assign a particular person as the only person who can do a particular step in a task**. Very linear, engineering kinds of approach to that. So they lay out all the steps of the task, they assign particular people to each step in the task. And then that person has to do that task and they have permissions to do that task that are limited. So it’s a little bit different than the way we sorta do it [in VA’s homegrown EHR]. We have some restrictions like only certain people can write orders. But theirs is much more prescribed as inherently built into their system. (ID13)

Councils were also involved in determining the nature and format of documentation that would be encouraged or required for each clinical team member. Many participants gave examples of ways that they established documentation templates which identify the kinds of information EHR users need to include to adequately document patient care, and the ways that, in doing so, the templates provide overt hints of what kinds of care ought to be provided and what patient information ought to be considered. One participant explained this decision-making in the context of otolaryngology visits: “We’re trying to work on an outpatient clinic visit. When somebody comes in, **when the screen comes up, how do you fill out that form? What does it automatically populate?** [For example,] medication lists and vital signs.” (ID12).

Another participant gave an example describing decisions their council made to develop documentation templates along with related recommended interventions:

That’s part of our directive in our mandate for care of our veterans with spinal cord injury, [which] can be a very life-threatening situation, and so we have to have this documentation. So our spinal cord injury national office leadership and subject matter experts from the field were pulled together … and **we created this document as well as the instructions, the order sets, for medication implementation for treatment as well as possible diagnostic tests**. … [For example,] if you have a patient with a spinal cord injury, interventions – physical interventions – have to be completed to regulate their bowels so they don’t have accidents. That was not [previously specified] in Cerner, and had to be created. (ID34)

### Why It Matters: Anticipated Implications of Standardization

We identified three key *implications* of national centralization: the potential for increased *efficiency* developing electronic tools and increased secondary uses; an accompanying increase in *bureaucracy* surrounding changes to the EHR; and increased *uniformity* of the care delivered across settings.

#### Efficiency

Participants indicated that national standardization of the EHR system involves standardizing the format of clinical data and standardizing the clinical processes that generate those data, which could yield nationwide data sets that are easier to interpret and require less work to aggregate. One participant described the potential implications of harnessing standardized data for quality improvement of emergency departments:

It’ll make it a lot **easier to do QA/QI** [quality assurance and quality improvement] projects looking at door-to-EKG time, looking at door-to-sepsis antibiotics time. Where, unless you have a really good data analytics person now, a lot of that work is manual in chart review. (ID41)

Participants also noted the potential for standardization to reduce duplication of effort across facilities — e.g., the work of configuring and implementing EHR-based tools (e.g., clinical decision support) would no longer need to happen independently at each of the VA’s many facilities. One participant described how this could help VA benefit from economies of scale and enable facilities’ EHR content to be simultaneously updated when medical knowledge evolves: “We were able to complete a **customized template** … that can be utilized … **across all settings**.”(ID34) This participant then went on to describe how this arrangement could enable *all* VA facilities’ EHR content to be updated as medical knowledge evolves, while simultaneously alluding to the increased work required to make national-level changes:

If anything should change from the literature and the research on how we intervene or implement our medication management … **it’ll take a national change for us to update it**. [The template is] not something that Cerner has had on their base document. Nobody’s ever had it. This is a one-of-a-kind creation. So that’s one of the perks. I mean, that’s one of the good things that’s come out of this. (ID34)

Another participant explained the economies of scale even more directly, while noting a trade-off of increased bureaucracy:

**Things that somebody might be able to just do quietly in an office now [will require] a lot of governance and preparation**, and in trade for doing that governance and preparation, which takes more time, **you do it once and it handles all of the sites**. (ID41)

#### Bureaucracy

This perspective, that standardization can be efficient but demands much greater governance, was widely held, and participants suggested that standardization could lead to the proliferation of veto points that make needed changes slow to occur and overly burdensome to pursue: “**My concern is that we’ll bureaucratize everything**, and we’ll put change control processes into place that are **so rigid that we won’t be able to really modernize** very well.”(ID27) One participant offered the example of hurdles they faced in getting desired content incorporated in the EHR:

We tried to have an order set for epidurals, we tried to have an order set for regional anesthesia, we tried to have order sets for Ketamine for pain, we tried to have an anesthesia note. [We] gave them the five little notes that we use and … **in the current VA system, it would take the current VA techs, oh, a day or so to make these five little notes. In three years, we haven’t seen one** from Cerner. (ID19)

Another participant reflected on the implications for those “current VA techs” and other local informaticists who, historically, have been able to build and implement EHR content at the request of local clinicians with relatively little national oversight:

As you transition to a system like Cerner, **a lot of the distributed field work is, in fact, centralized**. Decisions that needed to be done [at] the individual at every VA medical center start to get centralized. But **that doesn’t mean that we need less people. It just means that the authority of those people becomes centralized**. (ID41)

This participant conveyed the scope of standardization and its associated bureaucracy by noting that in addition to standardization among VA facilities, VA would also have to align much of its EHR content with Defense Health Agency facilities: “We also have, you know, the **absolutely necessity to coordinate things with the Department of Defense**—that we have never done before—because we [will be] sharing their drug database.” (ID41).

Participants also reflected on the ways that standardization-related bureaucracy could make it more difficult for facilities to meet local needs. One emphasized that, “**places are staffed very differently and structurally differ** … [A single standard] can go into a place that doesn’t have the structure to support the way you’ve designed the workflow [and] it’s gonna crash. It’s not gonna work.” (ID13) Another participant gave an example of important heterogeneity across sites by describing valid regional differences that could be difficult to anticipate and accommodate:

**How I prescribe … might be slightly different based on regional differences on how medicine is practiced**. So… here in [City A] we don’t have a high rate of tuberculosis – In fact, I think there’s actually been [only] one case documented to the health department in the last five years. Where I used to practice in [City B], it was endemic. We would regularly have patients admitted with tuberculosis infections. So, for us, we had a much different antibiotic diagram paying attention to the stuff around tuberculosis then is present here in [City A]. That’s a regional difference. (ID32)

#### Uniformity

Finally, participants explained a foundational, almost definitional implication of standardization: by reducing variation in EHR content across facilities, the organization is expected to reduce variation in clinical processes and quality of care. One participant asserted that, “the council really needs to be in charge of making sure that we’re standardizing processes to the extent possible, in order to **ensure one uniform standard of care** for all of our patients.” (ID24).

Another participant highlighted the need to justify each change on its own merits: “You might have a site that goes, ‘I don’t want my content to look like this.’ We, nationally, had better be able to say, ‘Oh, but United States Preventive [Services] Task Force doesn’t say to do what you want it to say. It says this.’ … **We cannot afford to put in front of people guidance and then not be able to point to ‘here’s where it came from**, here’s why you’re supposed to do it.’” (ID13).

Others elaborated on what it means in practice for VA to have increased uniformity, bringing about improvement at low-performing facilities but potentially enforcing a lowest common denominator that constrains high-performing facilities:

You have to establish what that standard is and **you have to be careful at how you can establish a standard and it’s the highest level of practice but can be achieved at a smaller size [and less complex] facility** but also then not have a standard that would cripple a [more complex] high-acuity facility. So you have to keep all that in mind when you’re standardizing. [You need to] listen to folks that are like, ‘I don’t know if I can achieve that.’ And say, ‘Well, you’re gonna need to get there,’ or ‘What is going to be a place that we can say is our starting part and that we can build from there?’ (ID01)

## DISCUSSION

In this qualitative study, we found that the VA’s ongoing EHR transition involves important and widespread organizational changes that are easily obscured by the technical complexity and sheer scope of the transition. Consistent with reports from other EHR transitions,^14,19^ we found that the standardization of clinical practice was an intentional and far-reaching goal of the VA’s EHR transition, with technological change used as a conduit for substantive changes to organizational policy. We also identified key implications of this clinical practice standardization, as anticipated by the organizational actors closest to the standardization process. These implications include potential efficiency gains, with less duplicated effort across facilities, but also an expanded bureaucracy that could make it more difficult for facilities to meet local needs or make dynamic adjustments. Standardization was also described as a driver of increased uniformity of both clinical processes and quality of care, simultaneously reducing wanted and unwanted variation in care.

While the degree to which clinical practice is shaped by the EHR may be taken for granted at many large healthcare systems that have fully integrated the EHR into care, this phenomenon has been relatively underexplored, and this study addresses this gap by illustrating several of the ways in which the EHR shapes practices. The study also fills a gap in the literature on EHR transitions, by exploring standardization as one of the key organizational transformations occurring as part of a large-scale EHR transition. While our study has particular relevance for EHR transitions, our findings are also relevant to the increasingly common and controversial^20^ efforts to standardize care, whether by means of the EHR or other mechanisms.

Our findings regarding the scale and implications of clinical standardization suggest that the process of determining standards demands substantial investments of time and resources. The process should include, at a minimum, (a) understanding existing variation in practice, (b) identifying best available evidence on optimal practices, (c) ensuring proposed standard practices are feasible across settings, (d) communicating and justifying new practices to affected employees, and (e) evaluating impacts of standardized practices.

It is also important to understand our findings in the context of the VA as a dynamic, ever-changing system. The structure and authority of the clinical councils whose members we interviewed have now been dramatically revised. Furthermore, the EHR-based standardization effort is accompanied by a concurrent VA initiative that is standardizing the scope of practice for employees across facilities and across state lines, asserting the VA’s legal authority to pre-empt state restrictions and provide a more consistent experience among VA facilities for employees and Veterans alike.^21^ And the question of standardization at VA has deep and complex roots. In fact, the VA went about a purposeful effort to *decentralize* decision-making in the 1990s, as part of a much-lauded transformation.^22,23^ Thus, current standardization efforts represent somewhat of a “pendulum swing” back to centralized governance.

Our findings are consistent with scholarship on the challenges of creating systems that require consensus and coordination across diverse actors with different agendas and priorities. Most proximally, this includes the challenges faced by the United Kingdom’s National Health Service in its effort to adopt a national EHR system,^19,24,25^ but also includes long-established scholarship in computer science (e.g., “the mythical man-month”),^26^ political science (e.g., “veto players”),^27^ and economic philosophy (e.g., “the knowledge problem”).^28^

There are also limitations to our study. While we capture perspectives about standardization from organizational actors who are particularly well-positioned to identify their implications, the EHR transition is still in its early stages, and the full effects of standardization are not yet observable. Future research should document how these standardization efforts play out at the local level as diverse facilities implement the new, standardized EHR. This study focused on perspectives from national-level VA decisionmakers, and as such, does not include perspectives from the front-line clinicians directly exposed to the EHR transition in their own clinical practice. We will address this limitation in future research, and are conducting multiple studies focused on the perspectives of front-line clinicians during EHR transitions.

In conclusion, we found that the VA’s EHR transition is a project involving vast clinical standardization and that this standardization involves profound potential implications for how care is delivered. These findings should inform the VA’s continued standardization and EHR transition process, along with other large-scale EHR transitions and clinical standardization initiatives.

### Supplementary Information

Below is the link to the electronic supplementary material.

Supplementary file1 (DOCX 27 kb)

## Data Availability

The datasets generated and analyzed during this study are not publicly available because they contain information that could compromise research participant privacy. However, anonymized data extracts are available from the authors upon reasonable request.
