# Supplementary material for: Software is Policy: Electronic Health Record Governance and the Implications of Clinical Standardization
Source: J Gen Intern Med. 2023 Oct 5;38(Suppl 4):949–55. doi: 10.1007/s11606-023-08280-7 (PMC10593671; doi:10.1007/s11606-023-08280-7)
Supplement: Supplementary file 1 — Supplementary file1 (DOCX 27 kb) [file 11606_2023_8280_MOESM1_ESM.docx]

**Qualitative Interviews of Clinical Council Participants**

*Background*

1. Let’s start by talking briefly about your background.
   1. What is your role within the VA? How long have you been in that role?
   2. How long you have been with the VA?

As you know, our focus today is the ______ council.

*Council structure*

1. To start, can you walk me through the work that the _____ council does?
2. What are the different perspectives represented on the council?
   1. Probe: any missing perspectives / expertise?
3. I understand there are several workgroups within the council. What role do those workgroups play?
   1. Probe [workgroup lead/participant]: tell me about what happens in the workgroup
4. How would you describe the relationship between this council and the other councils?
   1. Probe: division of responsibilities across councils, related challenges
5. Does the council have any ties to VISN- or medical center-level groups?
   1. Probe: VA national offices? (e.g. program offices)
   2. Probe: other groups that the council interacts with as part of its charge?

*Council processes*

1. Can you walk me through the things that happened in the national workshops?
   1. Probe: is there anything you would change about those workshops?
2. Then, I understand that there have also been ongoing meetings after the workshops. Can you describe what happens in those ongoing council and workgroup meetings?
3. [Time permitting] How has COVID affected the work of the council and workgroups?
4. How do you expect the role of the council to evolve over the next few years?

*Council outputs*

1. What types of clinical content are produced by the council? What are the products of its work?
   1. [Time permitting] How are products disseminated?
2. We’re interested in the different kinds of information and sources that the council considers when developing clinical content…. things like clinical guidelines, existing VA processes, and the individual experience and expertise of subject matter experts. Can you talk about what informs the clinical content?
3. Probe:
   - 1. VA policies/directives
     2. Workflow maps from VA site visits
     3. CPRS or DoD EHR content
4. How does the council prioritize clinical topics or workflows?
   1. Probe: clinical breadth / comprehensiveness
5. Is EHR usability taken into consideration?

*General*

1. Are there any lessons learned from your experience on the council that you would give as advice for other councils?
2. [Time permitting] If you could design the councils in any way that you wanted, what would that look like?
3. Who else do you recommend we talk to?
